# Integrating In Vitro Bioactivities and In Silico Molecular Evaluation of *Tamarix gallica* from Western Algeria

**DOI:** 10.3390/molecules31122168

**Published:** 2026-06-20

**Authors:** Fatima Kerroum, Salima Douichene, Fatiha Ben Ahmed, Aida Bassedik, Abdeslam Mohamed Dems, Manel Terbeche, Antoni Szumny

**Affiliations:** 1 Biotechnology and Agriculture Division, Biotechnology Research Center (C.R.Bt), Ali Mendjeli, New city UV 03, BP E73, Constantine 25000, Algeria or f.kerroum@crbt.dz (F.K.); m.dems@crbt.dz (A.M.D.); 2 Department of Biology, Laboratory of Pharmacognosy Api Phytotherapy, University of Abdel Hamid Ibn Badis, Mostaganem 27000, Algeria; itadz@yahoo.fr; 3 Department of Biology, Faculty of Life and Natural Sciences, University of Oran1, Ahmed Ben Bella, 1524 EL MNaouer, Oran 31000, Algeria; biochimie88@gmail.com; 4 Agronomy and Nutritional Sciences Department, Dr. Moulay Tahar Saida University, Saida 20000, Algeria; basseddik.aida@univ-saida.dz; 5 Laboratory of Structure, Elaboration and Application of Molecular Materials, Department of Biology, Faculty of Natural and Life Sciences, Mostaganem University, National Road 11 Kharouba, Mostaganem 27000, Algeria; 6 Laboratory for Isolation and Analysis of Bioactive Compounds, Department of Biocatalysis and Food Chemistry, Faculty of Biotechnology and Food Science, Wrocław University of Environmental and Life Sciences, Norwida 25, 50-375 Wroclaw, Poland

**Keywords:** *Tamarix gallica*, GCMS analysis, phenolic compounds, antioxidant activity, antidiabetic activity, anti-Alzheimer, molecular docking

## Abstract

The genus *Tamarix* L. includes several species widely used in traditional medicine for their therapeutic properties. This study aims to evaluate the bioactive potential of *Tamarix gallica* extracts from Western Algeria using an integrated in vitro and in silico approach. GC–MS analysis with BSTFA derivatization was performed to characterize the chemical profile of the methanolic fraction. In addition, total phenolic, flavonoid, and tannin contents were determined in methanolic extracts of leaves and stems. The biological activities were assessed using antioxidant (DPPH, ABTS, β-carotene, FRAP, O-phenanthroline, and cupric reducing assays), antimicrobial, antidiabetic, and anti-Alzheimer in vitro assays. Molecular docking was conducted to evaluate the inhibitory potential of selected flavonoids against α-amylase, acetylcholinesterase, and butyrylcholinesterase. Results revealed a rich metabolite profile dominated by long-chain aliphatic alcohols (including hentriacontan-12-ol), phytosterols (β-sitosterol), fatty acids, phenolic derivatives, and sugar alcohols. The extracts exhibited strong antioxidant activity (IC_50_ = 1.34 ± 0.43 and 12.32 ± 0.36 μg·mL^−1^), significant antimicrobial effects against the tested pathogens, and notable antidiabetic and anticholinesterase activities (IC_50_ = 78.65 ± 1.43 and 98.37 ± 1.07 μg·mL^−1^). Molecular docking analysis supported these findings, showing strong binding affinities of quercetin and rhamnetin toward the target enzymes. Overall, *T. gallica* exhibits promising multifunctional bioactivities with potential pharmaceutical relevance.

## 1. Introduction

Enzyme inhibition has become a common strategy in drug development, providing effective approaches for the development of new treatments for various human diseases. Several enzymes have been identified as therapeutic targets due to their roles in disease prevention or management, such as acetylcholinesterase (AChE) related to Alzheimer’s disease, and α-amylase, implicated in diabetes mellitus [1]. Plant secondary metabolites, produced as part of their defense mechanisms, are associated with the therapeutic properties of many plants [2]. Numerous naturally occurring plant compounds with antioxidant properties have been identified [3].

Quercetin and its methylated derivative, rhamnetin, are flavonoids widely recognized for their significant biological activities, particularly in the context of oxidative stress-related disorders [4]. Quercetin exhibits potent antioxidant activity through the direct scavenging of reactive oxygen species and the modulation of endogenous antioxidant defense systems [4]. In addition, growing evidence suggests that quercetin exerts antidiabetic effects by regulating glucose metabolism, enhancing insulin sensitivity, and inhibiting key carbohydrate-digesting enzymes, such as α-amylase and α-glucosidase. [4,5]. Furthermore, quercetin has demonstrated neuroprotective effects against Alzheimer’s disease, including the inhibition of amyloid-β aggregation, the reduction in neuroinflammation, and protection against oxidative neuronal damage [6,7]. Rhamnetin, a structurally related O-methylated flavonol, also exhibits notable antioxidant and anti-inflammatory activities and is increasingly recognized for its potential neuroprotective and metabolism-regulating effects, although it has been less extensively studied than quercetin [8]. These reported bioactivities highlight the potential of these compounds as promising candidates for the management of diabetes and neurodegenerative diseases.

Antibiotic resistance in some strains has increased research into natural antimicrobial agents derived from microorganisms [9] and plants [10]. These natural compounds can inhibit bacterial and fungal growth and may also induce microbial cell death [11]. In Algeria, several halophytic plants with recognized medicinal applications occur, notably *Tamarix gallica*. It belongs to the genus *Tamarix*, which comprises nearly 75 species [12]. In addition, it is a widespread tree forming woodlands in the western Mediterranean Basin, occurring in saline habitats such as salt marshes, ravines, and areas with brackish rivers [13]. These species are known for their traditional use in the treatment of various liver disorders, as well as for their hepatoprotective and stimulant properties. Leaf and flower infusions exhibit anti-inflammatory and antidiarrheal activities [14], as well as antiproliferative effects against Caco-2 colon cancer cells [15]. In addition, previous studies have reported a relationship between antimicrobial activity and bioactive compounds, such as phenolics, present in extracts of *T. gallica* [16].

Although *T. gallica* has been investigated in several international studies for its potential antidiabetic activity, particularly through α-amylase and α-glucosidase inhibition, as well as its neuroprotective potential against Alzheimer’s disease via inhibition of amyloid-β aggregation, these findings are based on non-Algerian samples [17,18]. In addition, experimental studies have demonstrated that *T. gallica* extracts can attenuate Alzheimer’s disease-like pathology and ameliorate cognitive impairments in animal models, further supporting their neuroprotective potential [19]. However, research in Algeria to date has primarily focused on the phytochemical composition and antioxidant properties of the species, without evaluating its neuroprotective or antidiabetic effects [20]. Furthermore, studies have shown that the biological activities of *T. gallica* vary with geographical origin, highlighting the importance of site-specific investigations [21].

Therefore, the lack of targeted pharmacological studies on *T. gallica* from Algeria, particularly from western regions, represents a significant research gap and highlights the need for further investigation into its potential neuroprotective against Alzheimer’s disease and antidiabetic activities.

In light of the limited pharmacological data on Algerian *T. gallica*, particularly from western regions, this study aims to evaluate its bioactive potential using an integrated experimental and in silico approach. Specifically, gas chromatography–mass spectrometry (GC–MS) analysis with N,O-bis(trimethylsilyl)trifluoroacetamide (BSTFA) derivatization was performed, along with the determination of total phenolic, flavonoid, and tannin contents in methanolic extracts of leaves and stems, as well as the evaluation of their antioxidant, antidiabetic, anti-Alzheimer’s, and antibacterial activities through in vitro assays. Additionally, molecular docking was performed to explore the interactions of selected compounds with acetylcholinesterase (AChE), butyrylcholinesterase (BChE), and α-amylase, followed by absorption, distribution, metabolism, excretion, and toxicity (ADMET) analysis to assess their pharmacokinetic and safety profiles. This approach aims to highlight *T. gallica* as a potential source of bioactive compounds for the management of oxidative stress-related and neurodegenerative disorders.

## 2. Results

### 2.1. GC-MS Analysis with BSTFA Derivatization

**Figure 1:**
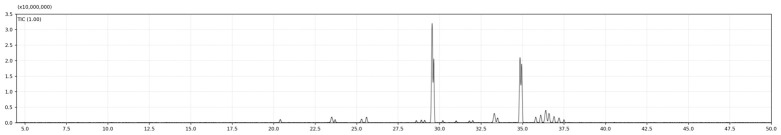
Representative chromatogram of *T. gallica* extract.

The methanol fraction of *Tamarix* spp. subjected to BSTFA derivatization revealed a diverse profile of polar and semi-polar metabolites detected as trimethylsilyl (TMS) derivatives. A total of 30 compounds were identified, representing 52.51 mg g^−1^ dry matter (d.m.). The major constituents were long-chain aliphatic alcohols, with hentriacontan-12-ol as the predominant compound (28.13 mg g^−1^), followed by 1-triacontanol (3.86 mg g^−1^) and 1-octacosanol (2.44 mg g^−1^). Other identified compounds included 5-henicosylresorcinol (0.53 mg g^−1^), β-sitosterol (4.97 mg g^−1^), squalene (1.28 mg g^−1^), palmitic acid (1.50 mg g^−1^), α-linolenic acid (1.32 mg g^−1^), and urolithin (0.35 mg g^−1^). Phenolic compounds such as trans-coumaric acid and trans-coniferyl alcohol were also detected. In addition, several sugars and sugar alcohols were identified, including tagatopyranose, arabinitol, galactose, glycerol, and erythritol. BSTFA derivatization enabled the detection of low-volatility polar metabolites present in the methanol fraction (Figure 1, Table 1).

### 2.2. Phenolic Composition of the Extracts

The total phenolic content (TPC) of *T. gallica* extracts was quantified using gallic acid as a standard. The calibration curve equation was y = 0.0083x + 0.0475 with an R^2^ of 0.9972, indicating a strong linear correlation within the detection range. Similarly, the total flavonoid content (TFC) was assessed using quercetin as the reference, with a calibration equation of y = 0.0048x and an R^2^ of 0.997. Results indicate that *T. gallica* leaves have significantly higher levels of total phenolics, 247.72 ± 0.03 µg Gallic Acid Equivalents mg^−1^ Extract (GAE); flavonoids, 49.65 ± 0.01 µg Quercetin Equivalents mg^−1^ E (QE); and condensed tannins (CTA), 78.43 ± 0.02 µg Catechin Equivalents mg^−1^ E (CE), compared to the stems (124.72 ± 0.26, 34.04 ± 0.05, and 56.96 ± 0.04 µg mg^−1^ E, respectively). This considerable difference indicates that the leaves are richer in secondary metabolites, which contribute to various biological activities (Table 2).

### 2.3. Antioxidant Properties of the Extracts

The antioxidant capacity of *T. gallica* was evaluated using six different assays based on distinct mechanisms: hydrogen atom transfer (2,2-diphenyl-1-picrylhydrazyl (DPPH) and β-carotene bleaching assays) and electron transfer-based methods, including 2,2′-azinobis(3-ethylbenzothiazoline-6-sulfonic acid) (ABTS), cupric ion reducing antioxidant capacity (CUPRAC), and ferric reducing antioxidant power (FRAP). The leaf extract showed notable radical scavenging activity in the ABTS assay (IC_50_ = 1.34 ± 0.43 μg·mL^−1^), nearly matching the synthetic antioxidant butylated hydroxyanisole (BHA) (IC_50_ = 1.29 ± 0.30 μg·mL^−1^) and surpassing α-tocopherol (IC_50_ = 34.93 ± 2.38 μg·mL^−1^). This suggests the ability of the extract to neutralize ABTS•+ radical cations through electron donation, likely due to phenolic acids and flavonoids. The FRAP assay (A_0.5_ = 12.32 ± 0.36 μg·mL^−1^) further confirmed its strong ferric-reducing power, attributed to polyphenolic compounds such as tannins. Conversely, the stem extract exhibited significantly weaker activity across all assays (e.g., ABTS IC_50_ = 23.23 ± 0.25 μg·mL^−1^), highlighting leaves as the main source of bioactivity. The DPPH assay (IC_50_ = 25.02 ± 0.32 μg·mL^−1^ for leaves) indicated moderate hydrogen-donating capacity, potentially influenced by solvent–system interactions in the assay medium. These mechanistic differences between electron-transfer and hydrogen atom transfer assays highlight the importance of using multiple methods to comprehensively evaluate antioxidant activity (Table 3).

### 2.4. Pearson Correlation Analysis

Pearson’s correlation analysis revealed positive relationships among TPC, TFC, and CTA, indicating that total phenolics, flavonoids, and condensed tannins followed a similar distribution pattern between leaf and stem extracts. Conversely, these phytochemical parameters were negatively correlated with the effective concentrations obtained in ABTS, DPPH, β-carotene bleaching, reducing power, phenanthroline, and CUPRAC assays. This inverse relationship is consistent with the fact that lower IC_50_ or A_0.5_ values reflect higher antioxidant effectiveness; therefore, extracts richer in phenolic constituents required lower concentrations to achieve the target antioxidant response. Accordingly, the leaf extract, which exhibited higher TPC, TFC, and CTA levels, showed markedly lower effective concentrations than the stem extract, confirming its stronger antioxidant potential. The strong positive correlations among antioxidant assays further support the internal coherence of the dataset, indicating that assays based on different reaction principles provided concordant discrimination between leaf and stem extracts. Overall, these findings suggest that the superior antioxidant performance of the leaf extract may be partly attributed to its higher content of phenolic compounds, particularly flavonoids and condensed tannins (Table 4).

### 2.5. Enzyme Inhibitory Activity of the Extracts

The results indicate that the leaf extract of *T. gallica* exhibits strong inhibition of α-amylase (IC_50_ = 78.65 μg·mL^−1^), showing higher activity than acarbose (IC_50_ = 3650.93 μg·mL^−1^), suggesting significant antidiabetic potential. Conversely, its inhibitory activity against AChE is moderate (IC_50_ = 124.15 μg·mL^−1^), while its effect on BChE is slightly stronger but still modest (IC_50_ = 98.37 μg·mL^−1^), indicating lower potency compared to synthetic inhibitors such as galantamine. The stem extract, in contrast, showed no inhibitory activity against AChE or BChE but demonstrated moderate inhibition of α-amylase (IC_50_ = 94.29 μg·mL^−1^), suggesting a weaker antidiabetic potential compared to the leaf extract. Overall, the leaf extract of *T. gallica* appears promising for antidiabetic applications, whereas the stem extract exhibits moderate activity and lacks cholinesterase inhibitory effects (Table 5).

### 2.6. Antimicrobial Potential of the Extracts

The methanolic extracts of *T. gallica* exhibited notable antimicrobial activity across a broad spectrum of microorganisms, as detailed in Table 6. Results indicate that the methanolic extract from *T. gallica* leaves is highly effective against all tested strains compared to stem extracts, with the strongest inhibition observed against *S. aureus* at 0.1 mg·mL^−1^. This finding is noteworthy given the increasing antibiotic resistance of *S. aureus*.

### 2.7. Molecular Docking Analysis of Flavonoid–Target Interactions

**Figure 2:**
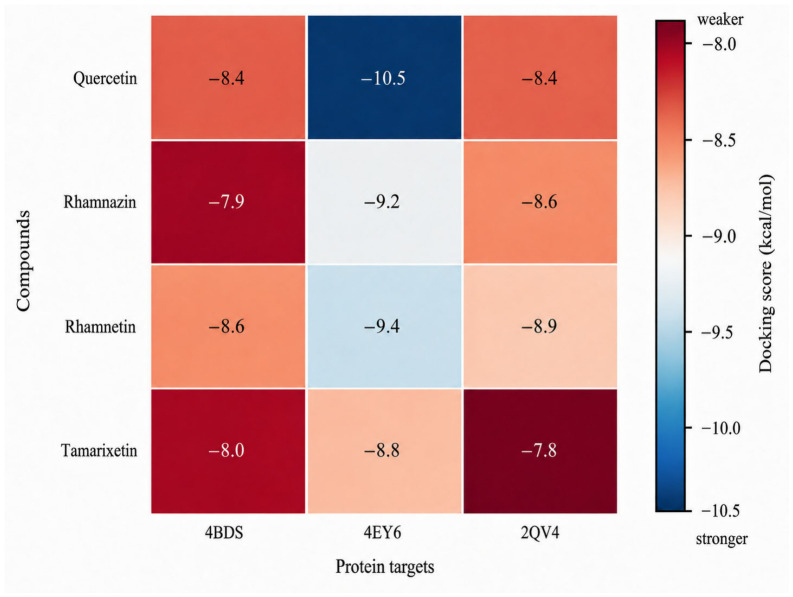
Heatmap of docking scores (ΔG).

**Figure 3:**
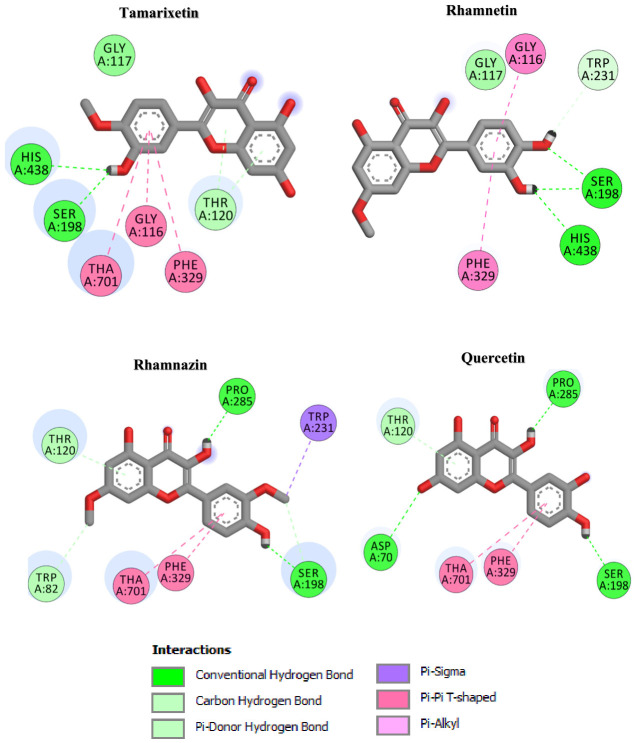
Binding interactions of the investigated compounds with human BChE (PDB ID: 4BDS).

**Figure 4:**
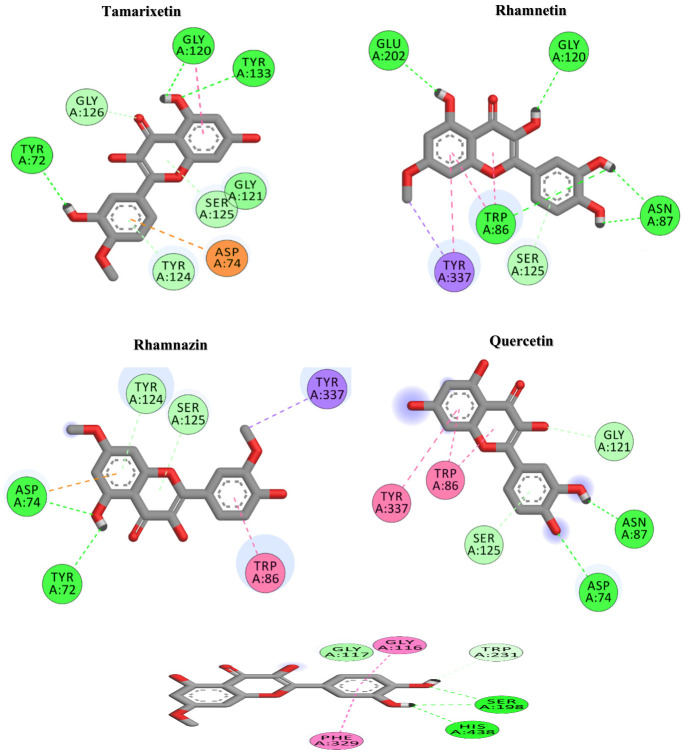
Binding interactions of the investigated compounds with human AChE (PDB ID: 4EY6).

**Figure 5:**
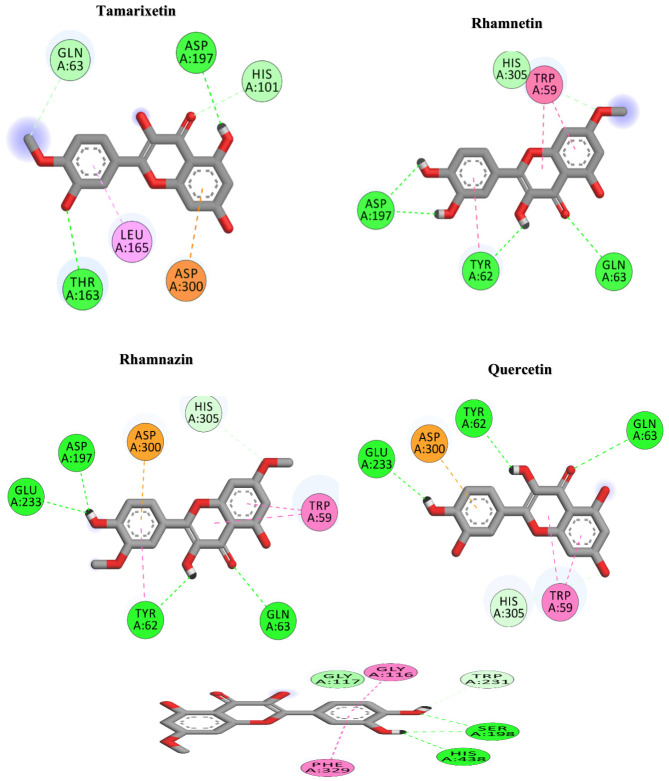
Binding interactions of the investigated compounds with human pancreatic alpha-amylase complexed with nitrite and acarbose (PDB ID: 2QV4).

Based on Figure 2, Figure 3, Figure 4 and Figure 5, quercetin exhibited the strongest inhibitory activity against AChE (4EY6), with a binding affinity of −10.5 kcal·mol^−1^, indicating the formation of a highly stable enzyme–ligand complex. This strong binding affinity may be attributed to the formation of multiple conventional hydrogen bonds with key residues in the catalytic gorge of the enzyme, including ASN87 and ASP74, as well as carbon–hydrogen interactions with GLY121 and SER125, in addition to strong π–π stacking interactions with TRP86 and TYR337.

The 2QV4 (α-amylase) complex indicated that quercetin exhibited a strong binding energy of ΔG = −8.4 kcal·mol^−1^, attributed to hydrogen bonds with TYR62, GLN63, and GLU233, as well as carbon–hydrogen interactions with HIS305 and π interactions with TRP59 and ASP300, thereby supporting its effective binding to the α-amylase active site.

In the BChE (4BDS) complex, quercetin exhibited a strong binding affinity (ΔG = −8.4 kcal·mol^−1^), primarily stabilized by hydrogen bonds with the catalytic residue SER198, as well as interactions with PRO285 and ASP70. Additional stabilization was provided by carbon–hydrogen interactions with THR120 and π–π interactions with PHE329, supporting a stable enzyme–ligand complex.

The inhibitory potential of rhamnetin was strong against both BChE (4BDS), AChE (4EY6), and α-amylase (2QV4). In the BChE (4BDS) complex, rhamnetin exhibited a strong binding affinity (ΔG = −8.6 kcal·mol^−1^), mainly stabilized by conventional hydrogen bonds with catalytic residues His438 and Ser198, π–π interactions with Phe329, and hydrophobic interactions with Trp231, contributing to stable binding within the catalytic gorge. In the AChE (4EY6) complex, rhamnetin showed a strong binding affinity (ΔG = −9.4 kcal·mol^−1^), supported by hydrogen bonds with catalytic residues Glu202, Gly120, Trp86, and Asn87, as well as π–π interactions with Tyr337, which further stabilized its binding within the catalytic gorge. Regarding α-amylase (2QV4), rhamnetin displayed the highest binding affinity among the tested compounds (ΔG = −8.9 kcal·mol^−1^), forming hydrogen bonds with catalytic residues Asp197, Tyr62, and Gln63, along with π interactions with Trp59 and van der Waals interactions with His305. Overall, rhamnetin exhibited excellent steric and electronic complementarity with cholinesterases and α-amylase, supporting its potential as a dual inhibitor of AChE, BChE, and α-amylase.

Tamarixetin showed relatively weaker inhibitory activity against AChE, BChE, and α-amylase. In the AChE (4EY6) complex, it exhibited a docking score of −8.8 kcal·mol^−1^, stabilized by hydrogen bonds with GLY120, TYR133, and TYR72, as well as carbon–hydrogen and π interactions with active-site residues such as ASP74 and SER125.

In BChE (4BDS), tamarixetin presented a moderate binding affinity with a ΔG value of approximately −8.0 kcal·mol^−1^, stabilized by hydrogen bonds with HIS438 and SER198, as well as π–π interactions with PHE329, indicating partial occupancy within the catalytic gorge. In α-amylase (2QV4), tamarixetin exhibited the lowest binding affinity (ΔG = −7.8 kcal·mol^−1^) among the tested flavonoids, with weak hydrogen bonding interactions involving ASP197 and THR163, which results in less favorable binding positioning within the active site of α-amylase.

### 2.8. In Silico ADME, Drug-Likeness, and Toxicity Evaluation

In silico analysis was performed to evaluate the physicochemical properties, pharmacokinetics, drug-likeness, and toxicity (Table 7) of quercetin and its corresponding methylated derivatives, rhamnazin, rhamnetin, and tamarixetin. Overall, all compounds exhibited acceptable profiles, with variations mainly dependent on the hydroxyl and methoxy functional groups involved in the modification of the flavonol structure.

The molecular weight of all molecules was less than 500 g·mol^−1^, with a Lipinski Rule of Five (Ro5) score below 5, indicating favorable oral bioavailability. The flavonol backbone of each molecule remained unchanged. The low number of sp^3^ carbon atoms and limited number of rotatable bonds revealed rigid planar structures, which may facilitate stable biological interactions.

Quercetin was the most polar compound, exhibiting the highest number of hydrogen bond donors and a topological polar surface area (TPSA) of 131.36 Å^2^. Methylation reduced compound polarity, with the lowest TPSA observed in rhamnazin (109.36 Å^2^), indicating improved membrane permeability. The logP values increased following methylation, ranging from 1.23 to 2.02. Water solubility remained acceptable for all compounds, except tamarixetin, which showed moderate solubility. All compounds were predicted to exhibit high gastrointestinal absorption and were not substrates of P-glycoprotein (P-gp). None of the flavonols were predicted to cross the blood–brain barrier, suggesting a reduced risk of central nervous system-related adverse effects. In contrast, all compounds were predicted to inhibit CYP1A2, CYP2D6, and CYP3A4, while rhamnazin and tamarixetin were additionally predicted to inhibit CYP2C9, indicating a potential risk of drug–drug interactions.

The compounds passed major drug-likeness filters, including Lipinski, Ghose, Veber, Egan, and Muegge rules, with a bioavailability score of 0.55. They were further evaluated for PAINS and Brenk structural alerts, where only quercetin and rhamnetin showed positive alerts, whereas rhamnazin and tamarixetin exhibited no structural toxicity alerts. Acute oral toxicity prediction indicated improved safety profiles for methylated derivatives, which were classified as Class V (LD_50_ = 5000 mg·kg^−1^), compared to quercetin (Class III, LD_50_ = 159 mg·kg^−1^). All compounds were predicted to be non-hepatotoxic and non-neurotoxic, suggesting an overall favorable safety profile.

In conclusion, the methylated derivatives of quercetin exhibited improved drug-likeness and safety profiles, supporting the selection of rhamnazin, rhamnetin, and tamarixetin for further molecular docking and pharmacological evaluation.

## 3. Discussion

The antioxidant properties of *T. gallica* methanolic extracts, evaluated through seven complementary assays, reveal significant radical scavenging potential. The strong ABTS activity of the leaf extract (IC_50_ = 1.34 ± 0.43 µg·mL^−1^), comparable to the synthetic antioxidant BHA (IC_50_ = 1.29 ± 0.30 µg·mL^−1^), highlights its ability to neutralize ABTS•+ radical cations through electron transfer mechanisms. This activity may be attributed to phenolic constituents such as trans-coumaric acid and trans-coniferyl alcohol, as well as bioactive compounds including squalene and β-sitosterol, which are known for their antioxidant and protective effects against oxidative stress [22]. This aligns with recent studies on *Tamarix* species, such as *T. aphylla*, where ABTS scavenging activity (IC_50_ = 1.8 µg·mL^−1^) was attributed to a high phenolic acid content [23]. Similarly, the FRAP results (A_0.5_ = 12.32 ± 0.36 µg·mL^−1^) suggest a notable ferric-reducing power, likely mediated by flavonoids and tannins, which are known to donate protons in hydrogen atom transfer reactions [24]. The superior performance of *T. gallica* over α-tocopherol (IC_50_ = 34.93 ± 2.38 µg·mL^−1^) underscores its potential as a natural alternative to lipid-soluble antioxidants.

The differing effectiveness of the assays (ABTS/FRAP/CUPRAC versus DPPH) may reflect differences in their reaction mechanisms. For example, ABTS, FRAP, and CUPRAC focus on electron transfer mechanisms, while DPPH depends mainly on hydrogen atom transfer [25]. This mechanistic difference highlights the importance of using multiple methods, as recently recommended [26] in studies on *Phoenix dactylifera* antioxidants. The strong correlation between phenolic content (Table 2) and antioxidant activity further supports the notion that polyphenols are the primary bioactive compounds, in line with findings for other Mediterranean medicinal plants [27].

In anti-enzymatic assays, the extract’s moderate inhibition of AChE and BChE, despite higher IC_50_ values than standards, aligns with trends observed in *Tamarix* spp. It could be related to the occurrence of phytosterols, particularly β-sitosterol, as well as antioxidant lipophilic compounds such as squalene. Phytosterols have been reported to exert neuroprotective effects by reducing oxidative stress, modulating amyloid-related pathways, and protecting neuronal cell membranes [28]. For example, *T. ramosissima* extracts exhibited AChE inhibition at IC_50_ = 45 µg·mL^−1^ [29], suggesting that *Tamarix* species may contain alkaloids or terpenoids with modest neuroprotective effects. In addition, the identification of urolithin is noteworthy because this metabolite and its derivatives are increasingly associated with neuroprotective, antioxidant, and anti-inflammatory activities. Urolithins have attracted considerable attention for their potential role in preventing neurodegenerative disorders and improving mitochondrial function [28]. Additionally, comparable anticholinesterase activity has been reported for other species of the same genus. Khodja et al. [30] demonstrated that the acetone seed extract of *T. africana* exhibited modest AChE inhibition with an IC_50_ = 102 μg·mL^−1^, a value consistent with our findings (IC_50_ = 124.15 μg·mL^−1^), confirming the limited but measurable cholinesterase inhibitory potential of *Tamarix* extracts. The differences observed across studies may be explained by variations in plant part, extraction solvent, geographical origin, and bioassay conditions, all of which are known to significantly influence the biological activity of plant extracts. Conversely, the notable α-amylase inhibition (IC_50_ lower than acarbose) indicates a unique antidiabetic potential, which may further be associated with the presence of β-sitosterol, unsaturated fatty acids, and squalene. Experimental studies have demonstrated that squalene exhibits antihyperglycemic effects and protective activity against oxidative stress associated with diabetes [31]. This strong α-amylase inhibitory activity is in agreement with previous reports highlighting its antidiabetic potential [17]. Notably, Nisar et al. [17] demonstrated significant inhibition of α-amylase and α-glucosidase by *T. gallica* extracts, which was attributed to their high phenolic content. These findings support the role of this species as a promising natural source for managing hyperglycemia.

The higher α-amylase inhibitory activity observed compared to acarbose may be attributed to the synergistic interactions of multiple bioactive compounds present in the extracts, particularly flavonoids and phenolic acids, rather than the action of a single molecule. Indeed, recent studies have demonstrated that combinations of phenolic compounds can exhibit enhanced inhibitory effects against α-amylase, often exceeding the activity of individual compounds or even standard drugs due to synergistic interactions at the enzyme level [32]. Similarly, flavonoids have been reported to act through multi-target and cooperative mechanisms, leading to enhanced enzyme inhibition when present as complex mixtures rather than as isolated constituents [33]. This synergistic behavior may explain the superior activity of the crude extract observed in the present study.

The difference in effectiveness between α-amylase inhibition and weaker cholinesterase inhibition may result from structural specificity: α-amylase has a larger active site capable of accommodating bulkier polyphenols, whereas AChE possesses a narrower gorge that may limit ligand interactions [34]. Additionally, synergistic effects from minor constituents (such as organic acids) could enhance α-amylase inhibition, as seen in *Rosmarinus officinalis* extracts [33,34].

Despite these promising results, the high concentrations needed for FRAP and anti-cholinesterase activities (e.g., A_0.5_ = 12.32 µg·mL^−1^) compared to synthetic drugs highlight a standard limitation of crude plant extracts. Future research should focus on bioassay-guided fractionation to isolate active compounds, as shown for *T. nilotica* anthocyanins, and assess in vivo efficacy to confirm their therapeutic potential [35].

The high inhibitory activity exhibited by quercetin and rhamnetin against α-amylase, AChE, and BChE in molecular docking studies can be attributed to their ability to form multiple hydrogen bonds through their hydroxyl groups, as well as their planar structures favoring π–π interactions with key residues such as TRP86 in AChE and PHE329 in BChE, as widely reported for flavonoid–cholinesterase interactions [34]. In addition, strong binding within the active site of α-amylase (2QV4) was observed, involving critical catalytic residues such as ASP197, GLU233, and ASP300, along with stabilizing interactions with TYR62, GLN63, and TRP59, which are known to play a key role in enzyme inhibition by flavonoids [36].

Quercetin exhibited the highest affinity due to the formation of multiple hydrogen bonds and π-interactions, while rhamnetin showed slightly reduced but still significant binding, consistent with reports indicating that flavonoids can act as effective dual inhibitors of α-amylase and other therapeutic targets [36]. Conversely, methoxylated derivatives such as rhamnazin and tamarixetin displayed reduced binding affinity, which can be explained by decreased hydrogen bonding capacity and steric hindrance, as methylation of hydroxyl groups has been shown to weaken α-amylase inhibitory activity [37]. Therefore, these flavonoids could serve as promising ligands for the development of novel therapeutic agents targeting both Alzheimer’s disease and diabetes.

The antimicrobial effects of the leaf extract are likely due to its rich content of bioactive compounds such as phenols, flavonoids, tannins, and saponins. These compounds are known to disrupt microbial membranes, interfere with essential enzymatic processes, and induce intracellular oxidative stress [38]. For instance, flavonoids and tannins can bind to bacterial and fungal cell membrane proteins, compromising their integrity and causing leakage of cellular materials, ultimately leading to cell death. Saponins can interact to lipid membranes, altering permeability and disturbing cellular homeostasis. The combined action of these compounds, along with their synergistic interactions, accounts for the leaf extract’s broad-spectrum antimicrobial activity [37].

The stem extract also demonstrated antimicrobial activity, but with slightly lower efficacy than the leaves. This may be attributed to its lower content of polyphenols and flavonoids, two classes of compounds that have been shown to exhibit strong antimicrobial activity in various studies [39]. The comparison between the two extracts suggests that *T. gallica* leaves are a more promising source of antimicrobial compounds, although the stems may still hold some potential for future research.

The antimicrobial activity of *T. gallica* methanolic extracts observed in this study could be linked to the abundance of long-chain aliphatic alcohols such as hentriacontan-12-ol, 1-triacontanol, and 1-octacosanol, together with alkylresorcinols and phenolic acids. Long-chain lipophilic alcohols are known to interact with microbial membranes, altering permeability and cellular integrity [40]. It aligns with recent findings on the pharmacological potential of *Tamarix* species. For instance, Alshehri et al. [41] reported dose-dependent antibacterial effects of *T. aphylla* stem extracts against Gram-negative bacteria, with inhibition zones of 12–14 mm at 1 mg·mL^−1^, which is consistent with our results against *P. aeruginosa* (15 mm) and *E. coli* (16 mm at 0.1 mg·mL^−1^). However, our study demonstrates notable efficacy at significantly lower concentrations (0.001–0.1 mg·mL^−1^), suggesting improved bioavailability of bioactive compounds in *T. gallica* or advancements in extraction methods. The superior activity of leaf extracts compared with stem extracts observed in the present study is consistent with their higher phenolic content, with leaves exhibiting a TPC of 247.72 µg GAE mg^−1^ compared to 124.72 µg GAE mg^−1^ in stems. Similar findings were reported by Aljubouri and Alobaidi [42] for *T. nilotica*, where differences in biological activity were attributed to variations in phenolic compound accumulation in plant tissues. The selective inhibition of *P. mirabilis* contrasts with weaker effects on *Salmonella typhi*, a pattern supported by Wang et al. [43], who attributed such differences to variations in bacterial efflux pump efficiency. Notably, the paradoxical increase in resistance of *Klebsiella pneumoniae* to polyphenol-rich extracts differs from recent reports. For example, the World Health Organization (WHO) [44] reported that polyphenols inhibit biofilm formation. In contrast, our results suggest potential adaptive resistance mechanisms, possibly involving stress-induced upregulation of efflux genes (e.g., acrAB-tolC). This discrepancy underscores the need for strain-specific mechanistic studies, particularly given the growing prevalence of multidrug-resistant *Klebsiella* strains [45]. The antifungal activity of *T. gallica* against *C. albicans* at concentrations of 0.1, 0.01, and 0.001 mg·mL^−1^ was greater than that of amphotericin B, which contrasts with earlier studies on *Tamarix* species [44]. This divergence may reflect differences in fungal strain susceptibility or extraction procedures. However, the highly effective concentrations observed also align with critiques regarding the practical limitations of crude plant extracts [45], emphasizing the importance of bioactive compound isolation. A novel aspect of this work lies in highlighting the potential influence of ecological conditions on the biological activities of the studied species. The observed bioactivity may be consistent with previous findings in *Artemisia annua*, where ecological conditions and plant–microbe interactions were shown to enhance the production of bioactive secondary metabolites [46]. Although these factors were not directly investigated in the present study, they may partly explain the observed variations. This suggests a possible influence of environmental conditions on the bioactive potential of the studied species [47], providing a framework for optimizing the cultivation of medicinal plants. For example, nutrient-rich soils with high organic matter, combined with arbuscular mycorrhizal fungi (AMF) inoculation, could maximize bioactive compound yields in *T. gallica*, as suggested by Zhao et al. [48] for other medicinal species.

The superior performance of leaves over stems aligns with their higher phenolic content, consistent with studies on Mediterranean medicinal plants [49]. However, the relatively high concentrations required for specific activities (e.g., FRAP) highlight limitations in bioavailability, which is typical of crude extracts. Future work should focus on bioassay-guided fractionation to isolate active compounds (e.g., flavonoids, anthocyanins) and confirm their efficacy in vivo. These results suggest that *T. gallica* leaves are a promising natural source of antioxidants and α-amylase inhibitors, with potential uses in functional foods or diabetes management. However, further optimization is necessary to achieve therapeutic relevance.

## 4. Materials and Methods

### 4.1. Plant Material and Extract Preparation

The aerial parts (leaves and stems) of *Tamarix gallica* used in this study were collected in October 2019 from the western region of Algeria (Oran). The plant material was botanically authenticated at the Department of Biology (Botanical Laboratory), Ahmed Ben Bella University, Oran 1. In Algeria, this species is locally known as “Tarfa” (الطرفة). The collected samples were thoroughly washed, air-dried at room temperature, and subsequently ground into a fine powder using an electric grinder. The extraction of the powdered material (10 g) was carried out by cold maceration in a methanol/water (Sigma Aldrich, Steinheim am Albuch, Germany) mixture (80:20, *v*/*v*) under dark conditions with continuous mechanical stirring for 24 h. The resulting extract was filtered, and the solvent was removed under reduced pressure before storage for further analysis [50].

### 4.2. GC–MS Analysis After BSTFA Derivatization

The extracts obtained and evaporated as described in Section 4.1. were subjected to derivatization using BSTFA. Briefly, approximately 5 mg of each dried extract was transferred into a 1.5 mL reaction vial containing the internal standard, trans-resveratrol (>99%, Per Hansen Supplements, Warszawa, Poland), dissolved in anhydrous pyridine. Subsequently, 20 µL of BSTFA (Merck, Warszawa, Poland) was added to the mixture. All analyses were prepared in triplicate, and the derivatization reaction was carried out at 80 °C for 3 h using a heating block. The derivatized samples were then injected into the chromatographic system for analysis. The samples were analyzed using a Shimadzu GC–MS system (GCMS-QP2020, Shimadzu, Kyoto, Japan) equipped with a Zebron ZB-5 capillary column (30 m × 0.25 mm i.d., 0.25 µm film thickness; Phenomenex, Torrance, CA, USA). Data acquisition was performed in both SCAN and SIM modes at a scan rate of 3 scans s^−1^. In SCAN mode, the mass range was set from m/z 50 to 650. Helium was used as the carrier gas at a constant flow rate of 0.98 mL min^−1^ with a split ratio of 1:50. The injector temperature was maintained at 250 °C. The oven temperature program was as follows: initial temperature of 100 °C held for 1 min, increased at 5 °C min^−1^ to 190 °C, followed by an increase at 10 °C min^−1^ to 300 °C, with a final hold of 10 min. Compound identification was performed using three complementary approaches: (i) comparison of experimentally calculated retention indices (RIs) with those reported in the NIST20 library, (ii) comparison of the obtained mass spectra with NIST20 spectral data, and (iii) spectral deconvolution and chromatographic data analysis. The following software packages were employed for data processing and analysis: GCMS Solution (v. 4.20), Spectrus Processor (v. S55S41), and AMDIS (v. 2.73) [51].

### 4.3. Phenolic Compound Contents

#### 4.3.1. Total Phenolic Content

The TPC was determined spectrophotometrically (Optima, SP-3000, Tokyo, Japan) by the Folin–Ciocalteu (Sigma Aldrich, Steinheim am Albuch, Germany) method [52] using gallic acid (Sigma Aldrich, Steinheim am Albuch, Germany) as a standard and expressed in μg GAE mg^−1^ extract.

#### 4.3.2. Total Flavonoid Content

The TFC was based on the complexation of Al^3+^ and the flavonoids, using quercetin (Sigma Aldrich, Steinheim am Albuch, Germany) for calibration [53] and expressed in μg QE mg^−1^ extract.

#### 4.3.3. Condensed Tannins Assay

The CTA was performed applying vanillin (Sigma Aldrich, Steinheim am Albuch, Germany) in the acid medium method [54] using catechin (Sigma Aldrich, Steinheim am Albuch, Germany) as the standard and expressed in μg CE mg^−1^ extract.

### 4.4. Antioxidant Activity

#### 4.4.1. ABTS Cation Radical Decolorization Assay

The spectrophotometric analysis of ABTS+ scavenging activity was determined [55]. The ABTS+ working solution was prepared by mixing ABTS reagent (Sigma Aldrich, Steinheim am Albuch, Germany) (7 mM) with potassium persulfate (Sigma Aldrich, Steinheim am Albuch, Germany) (2.45 mM). Subsequently, 160 μL of reactive solution was combined with 40 μL of each sample diluted in methanol at various concentrations. After 10 min incubation in darkness, the absorbance was recorded at 734 nm. Inhibition percentage was determined by comparing each measurement to the blank (methanol). The activity was computed according to the formula:

(1) $$I\;(\%)=\frac{\left(A\;control-A\;sample\right)}{A\;control}\times 100$$

where: A is the absorbance.

#### 4.4.2. Cupric Reducing Antioxidant Capacity

The CUPRAC of the extracts was determined by the CUPRAC method [56] with minor modifications. In a 96-well microplate, the samples (40 µL) were combined with (10 mM; 50 µL) CuCl_2_, (7.5 mM; 50 µL) neocuproine solution and (1 M; 60 µL) ammonium acetate buffer (Sigma Aldrich, Steinheim am Albuch, Germany). The mixture reaction was incubated at room temperature for 60 min, and the values were read at 450 nm using a reagent blank as reference. The obtained values were calculated and reported as A_0.5_ (μg·mL^−1^).

#### 4.4.3. Reducing Power Assay (FRAP)

The extract’s reducing capacity was evaluated according to Oyaizu [57]. Briefly, 10 μL of each sample at various dilutions was mixed with 50 μL of 1% potassium ferricyanide (Sigma Aldrich, Steinheim am Albuch, Germany) and 40 μL of 0.2 M phosphate buffer (pH 6.6). After incubating the mixture, 50 μL of 10% trichloroacetic acid (Sigma Aldrich, Steinheim am Albuch, Germany) was added, followed by 40 μL of distilled water. Then, 10 μL of 0.1% ferric chloride solution was incorporated, and absorbance was measured at 700 nm. Ascorbic acid (Sigma Aldrich, Steinheim am Albuch, Germany) served as the positive control. The results are presented as IC_50_ (μg·mL^−1^), representing the concentration required to achieve 50% of the maximum absorbance.

#### 4.4.4. DPPH Radical Scavenging Activity

The DPPH assay was used to evaluate the free radical scavenging activity of *T. gallica* extracts, following the method by [2]. A fresh DPPH (Sigma Aldrich, Steinheim am Albuch, Germany) solution was prepared in methanol at 0.025 mg·mL^−1^. Then, 1.950 μL of this solution was combined with 50 μL of *T. gallica* extracts at concentrations of 0.1, 0.5, 1, 2, and 5 mg/mL. The negative control contained 50 μL of methanolmixed with 1.950 μL of DPPH. After a 30 min incubation in darkness at room temperature, absorbance was read at 517 nm with a spectrophotometer. Reference antioxidants included acetic acid, BHA, and butylated hydroxytoluene (BHT) (Sigma Aldrich, Steinheim am Albuch, Germany). The DPPH radical inhibition percentage was determined using the following formula:

(2) $$\mathrm{DPPH}\;(\%)=\frac{\left(A\;control\;-\;A\;sample\right)}{A\;control}\;\times 100$$

where A is the absorbance.

#### 4.4.5. β-Carotene Assay

This test followed a modified version of a previously established protocol [58]. First, 0.5 mg of β-carotene (Sigma Aldrich, Steinheim am Albuch, Germany) was precisely weighed and dissolved in 1 mL of chloroform (Sigma Aldrich, Steinheim am Albuch, Germany). Then, 200 µL of Tween 40 (Sigma Aldrich, Steinheim am Albuch, Germany) and 25 µL of linoleic acid (Sigma Aldrich, Steinheim am Albuch, Germany) were added to form a uniform lipid mixture. After removing the solvent under reduced pressure, 50 mL of oxygenated distilled water was added with vigorous stirring to create a stable emulsion. Absorbance of this emulsion was measured at 470 nm. A 160 µL sample of the β-carotene emulsion was combined with 40 µL of extracts at various dilutions. Absorbance was recorded at 470 nm at time zero (t_0_) and after 120 min (t120) of incubation. The antioxidant activity was expressed as IC_50_ values calculated using the following equation:

I%= 1 − ((AS (t0) − As (t120))/(Ac (t0) − Ac (t120)) × 100(3)

where A_S_ and A_C_ are the absorbance of the sample and the control, respectively.

#### 4.4.6. O-Phenanthroline Assay

A reaction mixture was made by combining 30 µL of methanol with 0.5% O-phenanthroline (Sigma Aldrich, Steinheim am Albuch, Germany), 30 µL of 0.2% FeCl_3_ solution, 110 µL of methanol, and 10 µL of the extract at different concentrations. The mixture was incubated for 20 min at 30 °C, after which its absorbance was measured at 510 nm, and the percentage inhibition was determined [59].

### 4.5. Enzymatic Inhibition Tests

#### 4.5.1. In Vitro Anti-Alzheimer Activity

Anticholinesterase activity was evaluated for both AChE and BChE enzymes (Sigma-Aldrich, St. Louis, MO, USA). In this assay, 10 µL of the plant extract or galantamine (Sigma-Aldrich, St. Louis, MO, USA) at various dilutions was mixed with 20 µL of enzyme solution (5.32 × 10^−3^ U for AChE or 6.85 × 10^−3^ U for BChE) and 150 µL of sodium phosphate buffer (PBS; 100 mM, pH 8.0). The mixture was incubated for 15 min at 25 °C. Subsequently, 10 µL of 5,5′-dithiobis(2-nitrobenzoic acid) (Sigma Aldrich, Steinheim am Albuch, Germany) (DTNB; 0.5 mM) was added, followed by the specific substrate to trigger enzyme activity: 10 µL of acetylthiocholine iodide (0.71 mM) for AChE or 10 µL of butyrylthiocholine chloride (0.2 mM) for BChE. Absorbance was measured at 412 nm, and the enzyme inhibition percentage was calculated using the following formula:

Inhibition (%) = [(E − S)/E] × 100.(4)

where E is the enzyme activity in the absence of the test sample, and S is the enzyme activity in its presence [60].

#### 4.5.2. In Vitro Anti-Diabetic Activity

α-Amylase inhibitory activity was evaluated using the iodine/potassium iodide (IKI) method with some modifications [61]. For the assay, samples (extracts and acarbose) were dissolved in methanol at different concentrations. Briefly, 25 μL of each sample was mixed with 50 μL of α-amylase (Sigma-Aldrich, St. Louis, MO, USA) (1 U) and incubated at 37 °C for 10 min. After incubation, 50 μL of a 0.1% starch solution was added. The reaction was stopped by the addition of 25 μL of 1 M HCl (Sigma Aldrich, Steinheim am Albuch, Germany), followed by 100 μL of IKI (Sigma Aldrich, Steinheim am Albuch, Germany) reagent. Absorbance was measured at 630 nm. The percentage inhibition was calculated using the following formula to determine the inhibitory effect.

(5) $$(\%)\;\mathrm{Inhibition}=\frac{\left(A\;control\;-\;\;A\;control\;blanc\right)\;-\;\left(A\;sample\;-\;A\;sample\;blanc\right)}{\left(A\;control\;-\;A\;control\;blanc\right)}\;\times 100$$

where (A): the absorbance values.

### 4.6. Antimicrobial Activity

In this study, five human pathogenic ATCC strains, including fungi, Gram-negative, and Gram-positive bacteria, were utilized: *Escherichia coli* (8739), *Staphylococcus aureus* (6538), *Enterococcus faecalis* (49452), *Pseudomonas aeruginosa* (27853), and *Candida albicans* (90026). All strains were initially cultured on Sabouraud chloramphenicol agar (Sigma Aldrich, Steinheim am Albuch, Germany) at 30 °C for 18–24 h (for fungi) and Mueller–Hinton agar (Sigma Aldrich, Steinheim am Albuch, Germany) at 37 °C for 24 h (for bacteria) and then transferred to fresh nutrient agar plates. These strains were obtained from the university’s microbiology laboratory in Tamanrasset, Algeria. To evaluate antimicrobial activity, the standard disk diffusion assay was used to determine the zones of inhibition. Disks were loaded with 10 μL of extract solutions (dissolved in DMSO (Sigma Aldrich, Steinheim am Albuch, Germany)) at various concentrations. After incubation at 37 °C for 18–24 h, inhibition zones were measured to assess activity, with appropriate controls (gentamicin, chloramphenicol, amphotericin B); the tests were performed in triplicate for reliability [62].

### 4.7. In Silico Molecular Docking

#### Docking-Based Insights into the Inhibitory Potential of Flavonoids

Molecular docking was performed using AutoDock 4.0 [63] and AutoDock Vina [64] to evaluate the inhibitory potential of selected flavonoids against AChE (PDB ID: 4EY6), BChE (PDB ID: 4BDS), and alpha-amylase (PDB ID: 2QV4). The flavonoid ligands were selected based on literature reports identifying the most bioactive and pharmacologically relevant compounds. Protein structures were prepared by removing water molecules and co-crystallized ligands, followed by the addition of polar hydrogens and partial charges. Ligands were geometry-optimized and treated as flexible molecules, and grid boxes were centered on the catalytic gorges. Binding affinities were reported in kcal.mol^−1^. Docking poses and ligand–enzyme interactions were analyzed using Discovery Studio Visualizer (BIOVIA Discovery Studio 2026). Pharmacokinetic and drug-likeness properties were predicted with SwissADME [65], while toxicity profiles were assessed using ProTox-3.0 [66]. Structure–activity relationship (SAR) analysis was performed to relate binding affinities to key structural features of the flavonoids.

### 4.8. Statistical Analysis

All experiments were performed in triplicate (n = 3). Results are shown as mean ± standard deviation. Statistical analysis was carried out using one-way ANOVA with Tukey’s post hoc test for multiple comparisons, utilizing GraphPad Prism 5 and SPSS (version 23). A *p*-value below 0.05 was deemed statistically significant.

## Figures and Tables

**Table 1:** GC–MS profile of BSTFA-derivatized metabolites from *T. gallica* methanolic extract.

KI	Peak Name	tR (min)	mg·g^−1^ d.m.
1	Octanoic acid, 1258/1242	7.867	0.01
2	Glycerol, 1265/1254	8.008	0.35
3	Erythritol, 1501/1512	13.375	0.14
4	Arabinitol, 1725/1720	18.15	0.13
5	4-Coumaric acid, 1785	19.558	0.08
6	Tagatofuranose, 1800/1801	19.883	0.04
7	Tagatopyranose, 1824/1828	20.342	0.78
8	Neophytadiene, 1828/1832	20.525	0.37
9	Myristic acid, 1841/1850	20.683	0.13
10	Neophytadiene unknown isomer	21.233	0.10
11	Galactose, 1912/19	21.925	0.04
12	trans-Coniferyl alcohol, 1940/1936	22.058	0.09
13	Pentadecanoic acid 1950/1946	22.183	0.01
14	Palmitic Acid 2044/2049	23.467	1.50
15	Linoleic acid, 2207/2202	25.292	0.42
16	alpha.-Linolenic acid, 2210/2217	25.358	1.32
17	Stearic acid, 2239/2243	25.617	0.59
18	Arachidic acid, 2443/2436	27.425	0.16
19	1-Monopalmitin, 2578/2606	28.592	0.32
20	Urolithin, 2695/2692	29.325	0.35
21	Squalene, 2827/2820	30.042	1.28
22	Nonacosane, 2900/2900	31.058	0.83
23	Hentriacontane 3100/3100	32.958	0.79
24	1-Octacosanol 3134/3143	33.367	2.44
25	Hentriacontan-12-ol, 3248/3248	34.875	28.13
26	5-Henicosylresorcinol, 3274/3272	35.275	0.53
27	1-Triacontanol, 3330/3333	36.117	3.86
28	β-sitosterol, 3346/3348	36.392	4.97
29	unknown, 3361	36.608	2.29
30	1-Dotriacontanol, 3531/3537	40.067	0.46
			52.51

**Table 2:** Phenolic compound contents of *T. gallica* (μg mg^−1^ E).

Sample	TPC	TFC	CTA
Leaf Extract	247.72 ± 0.029 ^a^	49.65 ± 0.01 ^a^	78.43 ± 0.02 ^a^
Stem Extract	124.72 ± 0.26 ^b^	34.036 ± 0.05 ^b^	56.96 ± 0.04 ^b^

Values are expressed as mean ± SD (n = 3). Different superscript letters (a, b) within the same column indicate significant differences at ^a^ (*p* < 0.05) and ^b^ (*p* < 0.01).

**Table 3:** Antioxidant activity of *T. gallica* extracts (μg mL^−1^).

		A_0.5_			IC_50_	
Samples	Phenanthroline	CUPRAC	FRAP	ABTS	β-Carotene	DPPH
Leaf Extract	19.30 ± 0.62 ^a^	40.08 ± 1.19 ^a^	12.32 ± 0.36 ^a^	1.34 ± 0.43 ^a^	63.26 ± 1.69 ^a^	25.02 ± 0.32 ^a^
Stem Extract	41.54 ± 0.23 ^d^	78 ± 0.34 ^d^	32 ± 0.43 ^d^	23.23 ± 0.25 ^d^	82.98 ± 0.08 ^d^	43.26 ± 1.16 ^d^
BHT *	0.93 ± 0.07 ^b^	5.35 ± 0.71 ^b^	nt	1.29 ± 0.30 ^b^	0.91 ± 0.01 ^b^	6.14 ± 0.41 ^b^
BHA *	2.24 ± 0.17 ^c^	8.97 ± 3.94 ^c^	nt	1.81 ± 0.10 ^c^	1.05 ± 0.03 ^c^	12.99 ± 0.41 ^c^
Ascorbic acid *	nt	nt	6.77 ± 1.15 ^b^	nt	nt	nt
α-Tocopherol *	nt	nt	34.93 ± 2.38 ^c^	nt	nt	13.02 ± 5.17 ^d^

* Standard compounds, nt: not tested. Values are expressed as mean ± SD (n = 3). Different superscript letters (a–c) within the same column indicate significant differences at a (*p* < 0.05), b (*p* < 0.01), and c (*p* < 0.001), d (*p* < 0.0001).

**Table 4:** Pearson’s correlation between phenolic contents and antioxidant assays of *T. gallica* extracts.

Variable	TPC	TFC	CTA	ABTS	DPPH	β-Carotene	FRAP	Phenanthroline	CUPRAC
TPC	1								
TFC	1	1							
CTA	1	1	1						
ABTS	−1	−1	−1	1					
DPPH	−0.997	−0.997	−0.997	0.998	1				
β-carotene	−0.995	−0.995	−0.995	0.995	0.993	1			
FRAP	−0.999	−0.999	−0.999	1	0.998	0.993	1		
Phenanthroline	−0.999	−0.999	−0.999	1	0.997	0.994	0.999	1	
CUPRAC	−0.998	−0.998	−0.998	0.998	0.996	0.999	0.997	0.998	1

Values represent Pearson correlation coefficients calculated from individual replicate values of leaves and stem extracts.

**Table 5:** Antienzymatic activity of *T. gallica* (IC_50_ μg mL^−1^).

Scheme	Anti-AChE	Anti-BChE	Anti-Alpha-Amylase
Leaf Extract	124.15 ± 0.09 ^a^	98.37 ± 1.07 ^a^	78.65 ± 1.43 ^a^
Stem Extract	Na	Na	94.29 ± 0.67 ^c^
Galantamine *	6.27 ± 1.15 ^b^	34.75 ± 1.99 ^b^	-
Acarbose *	-	-	3650.93 ± 10.70 ^b^

* Standard compounds, Na: no activity. Values are expressed as mean ± SD (n = 3). Different superscript letters (a–c) within the same column indicate significant differences at a (*p* < 0.05), b (*p* < 0.01), and c (*p* < 0.001).

**Table 6:** Antibacterial activity extract of *T. gallica*.

Bacterial Strains	Leaf Extracts (mg·mL^−1^)			Stem Extracts (mg·mL^−1^)			Ampicillin (μg/disc)	Gentamicin (μg/disc)	Amphotericin B (μg/disc)
	0.1	0.01	0.001	0.1	0.01	0.001	10	10	20
*E. faecalis*	13 ± 0.0 ^b^	12 ± 0.0 ^b^	10 ± 0.0 ^c^	10 ± 0.0 ^c^	9 ± 0.00 ^d^	8 ± 0.00 ^d^	18 ± 0.01 ^a^	-	-
*P. aeruginosa*	15 ± 0.0 ^c^	14 ± 0.0 ^c^	12 ± 0.0 ^d^	12 ± 0.0 ^d^	11 ± 0.0 ^e^	9 ± 0.00 ^f^	18 ± 0.01 ^a^	20 ± 0.01 ^b^	-
*S. aureus*	20 ± 0.0 ^c^	17 ± 0.0 ^a^	15 ± 0.0 ^d^	16 ± 0.0 ^e^	14 ± 0.0 ^d^	1 0 ± 0.02 ^f^	17 ± 0.01 ^a^	20 ± 0.01 ^b^	-
*E. coli*	16 ± 0.0 ^b^	15 ± 0.0 ^b^	14 ± 0.0 ^c^	13 ± 0.0 ^c^	11 ± 0.0 ^d^	10 ± 0.01 ^e^	17 ± 0.01 ^a^	17 ± 0.01 ^a^	-
*C. albicans*	15 ± 0.0 ^a^	13 ± 0.0 ^b^	12 ± 0.0 ^c^	12 ± 0.0 ^c^	10 ± 0.0 ^d^	10 ± 0.01 ^e^	-	-	10 ± 0.02 ^d^

Inhibition zone diameters are expressed as mean ± SD (mm) (n = 3). Different superscript letters (a–c) within the same column indicate significant differences at a (*p* < 0.05), b (*p* < 0.01), and c (*p* < 0.001), d (*p* < 0.0001), e (*p* < 0.00001), f (*p* < 0.000001).

**Table 7:** Physicochemical characteristics, pharmacokinetics, drug-likeness, and toxicity prediction of quercetin, rhamnazin, rhamnetin, and tamarixetin.

Molecule	Quercetin	Rhamnazin	Rhamnetin	Tamarixetin
Physicochemical Properties				
Molecular weight (g/mol)	302.24	330.29	316.26	316.26
Num. heavy atoms	22	24	23	23
Fraction Csp^3^	0	0.12	0.06	0.06
Num. rotatable bonds	1	3	2	2
Num. H-bond acceptors	7	7	7	7
Num. H-bond donors	5	3	4	4
Molar Refractivity	78.04	86.97	82.5	82.5
TPSA (Å^2^)	131.36	109.36	120.36	120.36
Lipophilicity				
Log Po/w	1.23	2.02	1.63	1.85
Water Solubility				
Class	Soluble	Soluble	Soluble	Moderately soluble
Pharmacokinetics				
GI absorption	High	High	High	High
BBB permeant	No	No	No	No
P-gp substrate	No	No	No	No
CYP1A2 inhibitor	Yes	Yes	Yes	Yes
CYP2C19 inhibitor	No	No	No	No
CYP2C9 inhibitor	No	Yes	No	Yes
CYP2D6 inhibitor	Yes	Yes	Yes	Yes
CYP3A4 inhibitor	Yes	Yes	Yes	Yes
Drug-likeness				
Lipinski	Yes	Yes	Yes	Yes
Ghose	Yes	Yes	Yes	Yes
Veber	Yes	Yes	Yes	Yes
Egan	Yes	Yes	Yes	Yes
Muegge	Yes	Yes	Yes	Yes
Bioavailability Score	0.55	0.55	0.55	0.55
Medicinal Chemistry				
PAINS	1 alert: catechol A	0 alert	1 alert: catechol A	0 alert
Brenk	1 alert: catechol	0 alert	1 alert: catechol	0 alert
Lead-likeness	Yes	Yes	Yes	Yes
Synthetic accessibility	3.23	3.41	3.3	3.26
Oral toxicity				
LD50 (mg/kg)	159	5000	5000	5000
Class	3	5	5	5
Organ toxicity				
Hepatotoxicity	Inactive	Inactive	Inactive	Inactive
Neurotoxicity	Inactive	Inactive	Inactive	Inactive

TPSA: Topological polar surface area, BBB: Blood–brain barrier, GI: Gastrointestinal, P-gp: P-glycoprotein, CYP: Cytochrome P450 enzymes, Log Po/w: logarithm of the octanol/water partition coefficient, Csp^3^: fraction of sp^3^-hybridized carbon atoms.

## Data Availability

The data that support the findings of this study are available from the corresponding author on reasonable request.

## Abbreviations

The following abbreviations are used in this manuscript:

GC-MS	Gas Chromatography–Mass Spectrometry
BSTFA	N,O-Bis(trimethylsilyl)trifluoroacetamide
AChE	Acetylcholinesterase
BChE	Butyrylcholinesterase
ADMET	Absorption, Distribution, Metabolism, Excretion, and Toxicity
TPC	Total Phenolic Content
TFC	Total Flavonoid Content
CTA	Condensed Tannin Content
GAE	Gallic Acid Equivalents
QE	Quercetin Equivalents
CE	Catechin Equivalents
ABTS	2,2′-azino-bis(3-ethylbenzothiazoline-6-sulfonic acid)
CUPRAC	Cupric Ion Reducing Antioxidant Capacity
FRAP	Ferric Reducing Antioxidant Power
DPPH	2,2-diphenyl-1-picrylhydrazyl
BHA	Butylated hydroxyanisole
BHT	Butylated hydroxytoluene
IKI	Iodine–Potassium Iodide
SAR	Structure–Activity Relationship
